# Tumor Microenvironment: Cellular Interaction and Metabolic Adaptations

**DOI:** 10.3390/ijms25073642

**Published:** 2024-03-25

**Authors:** Monica Benvenuto, Chiara Focaccetti

**Affiliations:** 1 Department of Clinical Sciences and Translational Medicine, University of Rome “Tor Vergata”, 00133 Rome, Italy; 2 Departmental Faculty of Medicine, Saint Camillus International University of Health and Medical Sciences, 00131 Rome, Italy

The tumor microenvironment (TME) plays a critical role in cancerogenesis. Various components of the TME, such as cancer cells, cancer-associated fibroblasts (CAFs), endothelial cells (ECs), tumor-infiltrating immune cells, adipocytes, and the extracellular matrix (ECM), collectively influence cancer development, progression, and the adaptation of cancer cells, as well as their resistance to anti-cancer therapies [1,2].

The objective of this Special Issue, entitled “Tumor Microenvironment: Cellular Interaction and Metabolic Adaptations”, which includes one research article and four reviews, is to shed light on the contribution of the TME to cancer development and progression, and to present recent advances in the development of novel therapeutic approaches for treating metastatic tumors through TME remodeling.

Research has elucidated how different elements within the TME synergistically influence cancer development and progression. For instance, CAFs have been identified as key players in various stages of tumor development, promoting tumor growth, angiogenesis, invasion, and the immune evasion of cancer cells [3,4]. 

Moreover, the presence of immune effector cells in the TME plays a pivotal role in shaping tumor growth direction. The immune system is crucial in both the initial stages of tumor formation and cancer progression, serving a dual purpose within the TME. Initially, innate immune cells like innate lymphoid cells, neutrophils, natural killer (NK) T cells, γδ T cells, NK cells, and macrophages orchestrate an immune response, followed by cytotoxic (CTL, CD8^+^) and helper (T_H_, CD4^+^) T lymphocytes, to eliminate emerging cancer cells. These T cells produce interferon-γ (IFN-γ) upon recruitment into the TME, promoting cell death, activating NK cells and macrophages, stimulating the humoral response, and inducing inflammation [5,6,7]. However, this elimination process leads to immune selection and shaping, resulting in the development of less immunogenic cancer cell variants that are resistant to immune attack during the equilibrium phase. Additionally, tumors can evade immune system-mediated elimination by recruiting immunosuppressive leukocytes and soluble factors, creating a microenvironment that hinders the effectiveness of the antitumor immune response [1,8]. ECs actively participate in the TME by interacting with immune cells and secreting angiogenic substances like chemokines, growth factors, and cytokines. The crosstalk between the tumor endothelium and immune cells significantly impacts the immune response to tumors and contributes to immunosuppression. Furthermore, tumor-associated ECs aid in the growth of immunosuppressive cell types like Treg cells, assisting in the evasion of the tumor immune system. Additionally, the development of distant metastasis is directly influenced by tumor-associated ECs of lymphatic and blood arteries [9,10,11]. Metabolic dynamics and factors, such as hypoxia and inflammation, also play a role in shaping the tumor-supportive microenvironment. Hypoxia, characterized by oxygen deprivation, profoundly affects tumor biology, driving angiogenesis, tumor invasion, and therapy resistance. Intratumoral hypoxia is a characteristic feature of solid tumors. The paper by Jeon et al. discusses the functions of hypoxia and hypoxia signaling molecules in skin cancer cells. The well-known hypoxia-inducible factor (HIF) pathway is essential to the hypoxic response of skin cancer cells. This paper describes the main genetic changes linked to skin cancer, and how they relate to hypoxia signaling, highlighting the potential of hypoxia-targeted therapies in skin cancer management [Contribution 1].

Although many inhibitors were investigated, interfering with mRNA expression, protein synthesis and degradation, none of these agents were approved for the treatment of cancer patients, and the therapeutic efficacy of HIF inhibitors needs to be improved [12].

Pacifico et al. explore the intricate role of glutamine metabolism in controlling the fate of cancer stem cells (CSCs), particularly in solid tumors. Glutamine, an essential amino acid, is crucial to many metabolic pathways vital for the survival and function of CSCs. This review also discusses the interplay between CSCs and the TME, focusing on CAFs, adipocytes, and senescent cells, which indirectly influence CSCs’ fate by modulating glutamine availability [Contribution 2].

The review by Munkácsy et al. emphasizes the roles of CAF, TAM, tumor-infiltrating lymphocytes (TILs), cancer-associated adipocytes (CAA), tumor-associated neutrophils (TAN) and NK cells in the triple-negative breast cancer (TNBC) microenvironment. In addition, it explores the contribution of metabolic pathways and associated signaling in the metabolic reprogramming of TNBC cells, which accelerate tumor progression and could be possible targets for the diagnosis and treatment of TNBC patients [Contribution 3].

Colorectal cancer (CRC) represents a significant global health challenge, necessitating the development of improved treatments. Autophagy, along with apoptosis and inflammation, plays a crucial role in CRC development, influencing various cellular processes including survival, proliferation, differentiation and immune response. In the paper by Kasprzak et al., the role of the insulin-like growth factor (IGF) signaling pathway in the mechanism of autophagy in CRC is discussed. This review emphasizes the importance of investigating multiple cellular pathways in CRC to define more effective combined therapeutic strategies [Contribution 4].

The development of anti-cancer approaches aimed at remodeling the TME shows great promise. Inhibitors of the ubiquitin proteasome system (UPS), particularly of the proteasome, are gaining significance due to their role in regulating the immune response. For example, bortezomib has shown the ability to prolong the survival of mice bearing salivary gland adenocarcinoma and to reshape the TME by increasing the number of tumor-infiltrating immune cells (CD4^+^ and CD8^+^ T cells, macrophages, and/or NK cells) [13].

Using immune checkpoint inhibitors (ICIs) has emerged as a promising approach, harnessing the immune system to target cancer cells and identifying predictive biomarkers and factors influencing treatment outcomes. These approaches are critical for optimizing cancer patient management. A retrospective study investigating the prognostic implications of fat loss following ICI treatment in patients with metastatic clear cell renal cell carcinoma (ccRCC) is reported in the paper by Lee et al. The findings reveal a compelling association between subcutaneous fat (SF) loss and diminished overall survival (OS) and progression-free survival (PFS) in this patient population. Thus, identifying SF loss following ICI therapy as a prognostic marker for metastatic ccRCC holds potential clinical utility in risk stratification and treatment decision making [Contribution 5].

Another promising approach could be the use of combination therapies aimed at simultaneously modulating the TME and targeting different signaling pathways involved in cancer cell growth and invasion [14]. However, further studies are needed to fully understand the role of all components of the TME in tumor progression to develop novel effective strategies for cancer patients.

## Data Availability

Not applicable.
